# Views of Practitioners and Researchers on the Use of Virtual Reality in Treatments for Substance Use Disorders

**DOI:** 10.3389/fpsyg.2021.606761

**Published:** 2021-05-21

**Authors:** Rigina Skeva, Lynsey Gregg, Caroline Jay, Steve Pettifer

**Affiliations:** ^1^Department of Computer Science, Faculty of Science and Engineering, Advanced Interfaces-Visual Computing, University of Manchester, Manchester, United Kingdom; ^2^School of Health Sciences, Faculty of Biology, Medicine and Health, Division of Psychology and Mental Health, University of Manchester, Manchester, United Kingdom; ^3^Department of Computer Science, Faculty of Science and Engineering, Information Management, University of Manchester, Manchester, United Kingdom

**Keywords:** virtual reality exposure therapy, substance abuse, avatars, craving, coping, mental health, therapists, cognitive behavioral therapy

## Abstract

Virtual Reality Therapy (VRT) has been shown to be effective in treating anxiety disorders and phobias, but has not yet been widely tested for Substance Use Disorders (SUDs) and it is not known whether health care practitioners working with SUDs would use VRT if it were available. We report the results of an interview study exploring practitioners’ and researchers’ views on the utility of VRT for SUD treatment. Practitioners and researchers with at least two years’ experience delivering or researching and designing SUD treatments were recruited (*n* = 14). Interviews were thematically analyzed, resulting in themes relating to the safety and realism of VRT, and the opportunity for the additional insight it could offer to during SUD treatment. Participants were positive about employing VRT as an additional treatment for SUD. VRT was thought suitable for treating adults and people with mental health issues or trauma, provided that risks were appropriately managed. Subsequent relapse, trauma and over-confidence in the success of treatment were identified as risks. The opportunity VRT offered to include other actors in therapy (via avatar use), and observe reactions, were benefits that could not currently be achieved with other forms of therapy. Overall, VRT was thought to offer the potential for safe, realistic, personalized and insightful exposure to diverse triggering scenarios, and to be acceptable for integration into a wide range of SUD treatments.

## Introduction

Substance use disorders (SUDs) are a global health issue. In 2018, 31 million people were officially diagnosed with a SUD (World Health Organisation, 2018). Substance abuse also leads to increased unemployment, crime and homelessness (Barber et al., 2017), at an ultimate cost of $600 billion per year in the United States (National Institute on Drug Abuse, 2018) and £10.7 billion in the United Kingdom (Barber et al., 2017). Current alcohol abuse treatment typically involves planned withdrawal, in a hospital or residential setting for severe cases, followed by cognitive behavioral, social network or environment-based interventions, combined with medication when necessary (National Institute for Health and Care Excellence, 2011). Drug abuse treatment guidelines suggest pharmacological interventions for detoxification, commonly in a community-led or inpatient setting (National Institute for Health and Care Excellence, 2007b). Contingency management, behavioral couple’s therapy and family interventions are also used to support detoxification and prevent relapse. Psychodynamic or Cognitive Behavioral Therapy (CBT) is preferred for people with co-morbid mental health issues (National Institute for Health and Care Excellence, 2007a).

SUDs are often chronic relapsing conditions (National Institute on Drug Abuse, 2018) and as such treatments focus on coping with the temptation to relapse in high risk situations. One of the techniques to practice coping is through exposure to triggering situations or cues within a therapeutic context. Two approaches that involve this are Imaginal Exposure (IE), which is a part of CBT, and Cue Exposure Therapy (CET). Exposure to triggers takes place, in the first approach, via the imagination and, in the second approach, in reality. While visiting triggering situations is possible in IE, it lacks realism (Gregg and Tarrier, 2007). In contrast, realistic exposure to proximal cues (e.g., a package of cigarettes) happens in CET either via photographs or actual objects, yet experiencing contextual cues (e.g., a pub) raises issues of practicality and the risk of relapse (Mellentin et al., 2017). Previous research shows that CET has limited efficacy in the treatment of SUDs and that, in comparison, CBT offers better therapeutic outcomes (Martin et al., 2010; Mellentin et al., 2017).

Exposure to triggers using Virtual Reality (VR) may offer a flexible alternative to IE and CET for practicing coping. A standard VR set-up typically consists of the VR headset, two hand-held controllers and a laptop that runs the VR application. Triggering situations can be rendered in 3-dimensions via the VR headset, allowing exposure to realistic, complex environments as opposed to the exposure during CET which involves mainly proximal cues (Martin et al., 2010). The controllers enable navigation in the virtual environments (VEs) and interactions with the avatars and objects presented within. The multi-sensorial nature of virtual exposure to triggers therapeutically may also increase engagement and therefore its clinical effectiveness beyond that of IE, as seen in PTSD, flying phobia and panic and agoraphobia (Gregg and Tarrier, 2007; Schwartzman et al., 2012; Maples-Keller et al., 2017; Deng et al., 2019; Singh and Nathan-Roberts, 2019). Virtual exposure to triggers may be combined with diverse therapeutic contexts, resulting in different forms of Virtual Reality Therapy (VRT). The content of the VR application will depend on the therapeutic context each time. Experimental evidence has shown that in both social and interpersonal VEs craving was invoked successfully (Hone-Blanchet et al., 2014; Pericot-Valverde et al., 2019; Segawa et al., 2020), and VRT may thus be able to support people in coping with these situations in a controlled, safe, therapeutic setting.

Despite its potential, the efficacy of VRT in the treatment of SUDs has been explored by a small number of studies. So far, VRT has been considered for treating nicotine, alcohol, cannabis, cocaine and synthetic psycho-stimulants abuse after eliciting related craving effectively (Martin et al., 2010; Hone-Blanchet et al., 2014; Amista, 2017; Bordnick and Washburn, 2019; Grochowska et al., 2019; Pericot-Valverde et al., 2019; Trahan et al., 2019; Wang et al., 2019; Worley, 2019; Kim and Kim, 2020; Segawa et al., 2020; Simon et al., 2020). It has also been reported to facilitate moderate craving reduction, being employed in most studies in the context of CET, (Martin et al., 2010; Hone-Blanchet et al., 2014; Amista, 2017; Bordnick and Washburn, 2019; Grochowska et al., 2019; Pericot-Valverde et al., 2019; Trahan et al., 2019; Wang et al., 2019; Worley, 2019; Kim and Kim, 2020; Segawa et al., 2020) and with more consistent results for alcohol abuse than for other substances (Ghiţă and Gutiérrez-Maldonado, 2018; Ghiţă et al., 2019). These results have been produced primarily in pilot and feasibility studies, and more controlled clinical trials with larger samples, improved quality protocols (e.g., less sensitive craving measures, non-exposure to cues between the trials) and follow ups are necessary to better determine VRT’s efficacy (Martin et al., 2010; Hone-Blanchet et al., 2014; Amista, 2017; Ghiţă and Gutiérrez-Maldonado, 2018; Trahan et al., 2019; Kim and Kim, 2020; Segawa et al., 2020).

Surveys have revealed that practitioners hold positive views about its potential for use in clinical practice generally, in particular regarding its ability to provide exposure to hard-to-access situations and easier manipulation of relevant stimuli (Segal et al., 2011). Financial cost relating to the VR hardware and software, technical difficulties and the training required were the most widely identified barriers (Segal et al., 2011). A further survey in 2012 of 262 therapists who had not previously used VR, showed concerns about training, equipment, costs, and a lack of understanding of the benefits and applications of VR (Schwartzman et al., 2012). By 2019, a survey of 185 behavioral therapists reported lower concerns around technical and cost related issues, as VR equipment had been widely commercialized from 2016 (Lindner et al., 2019). Likewise, therapists with knowledge of VR and VRT reported they were more likely to use it in the future (Lindner et al., 2019).

VRT has shown promise as a treatment for SUD, but its capabilities for relapse prevention or other therapeutic purposes and its capabilities in therapeutic contexts other than CET have not been fully investigated. The primary aim of this interview study was to explore the views of practitioners who had not used VRT in SUD treatment about its potential. The results will be used to develop a new VRT application and protocol. By taking a qualitative approach, we provide a richer understanding of therapists’ opinions on VR in clinical practice, which will help to refine the development of VRT as a treatment for SUD.

## Materials and Methods

### Participants

Psychologists, psychotherapists, counselors, social workers, NHS nurses and academics with an expertise in SUD treatment were interviewed. The sampling was purposive and the inclusion criterion was at least 2 years’ experience delivering or researching and designing substance abuse treatment. University and SUD counseling and recovery organization websites in the North West of England were searched to identify possible participants. Participants were invited to a face-to-face interview via the email address that appeared on the university or counseling website or the email address provided by the charities or the rehabilitation service they worked for, after initial contact with their administration center. Private counseling practices, a university, two charities and a rehabilitation and integration service were involved in the study.

### Procedure

Informed consent was given by all participants. Each participant was interviewed once at their place of work by a single researcher (first author). The interview started with a brief introduction to VR and VRT. This covered its application to phobias and PTSD, providing examples from literature, but its application to SUD was briefly mentioned as per the exposure it could offer to triggering situations, to avoid participants having any preconceptions about its possible contexts, delivery methods and content. Images were used to showcase a commercial VR headset with two hand-held controllers, its standard set-up and the view that the wearer will obtain when experiencing VR and when interacting with virtual objects.

For the interview, a semi-structured format was used for in-depth exploration of the thoughts of the experts in an open-ended manner (McIntosh and Morse, 2015; DeJonckheere and Vaughn, 2019). The topic guide of the interview involved questions about the acceptability of VRT and more specifically about the risks, challenges, benefits and conditions for using VRT in daily, clinical practice. Aspects such as the target population and eligibility criteria, delivery protocols and contexts, and preferences on the VR application content were further discussed. Two images of avatars applying social pressure from previous VRT studies in alcohol abuse were also given to the participants when asked about the VR application content, to offer a tangible example of what avatars would potentially look like. All interviews were audio-recorded, transcribed verbatim and anonymized, except for one interview where the encrypted digital recorder failed to save the recording. In this case the handwritten notes of the researcher were anonymized and used in the analysis. The content of the notes was checked by the participant for accuracy. Each interview lasted between 45 and 80 min. No compensation was provided for participating in the study.

### Thematic Analysis

Thematic analysis was chosen as the acceptability of VRT in SUD is a novel research topic and an analytical approach was needed (Braun and Clarke, 2006). The themes were identified using an inductive method, with the data driving their formulation. Furthermore, the identified themes were semantic (Braun and Clarke, 2006) as the explicit meaning of the data was analyzed. Consequently, the impact of socio-cultural or any other factors on the participants’ opinions was not considered as per the realist approach (Braun and Clarke, 2006).

The analysis followed the steps outlined by Braun and Clarke (2006). The first author read the transcripts repeatedly to promote familiarization with their content. During the formulation of the primary codes, segments of the transcripts were grouped into relevant concepts-codes. Coding was supervised throughout, and once all transcripts had been coded, the result was shared with the other researchers for validation. To further refine the codes, this process was repeated until each code was efficiently represented by the extracts attached to it. Then, the themes were identified and discussed with the research team. Some codes were not included, as they seemed irrelevant to the themes and could not form individual, meaningful themes. The coding was terminated once the themes were validated by the other researchers. The naming of the themes was crystallized once the thematic analysis report was written.

### Ethical Approval

Ethical approval for the study was granted by the University of Manchester Research Ethics Committee (Ref.: 2019-6983-10288).

## Results

### Participants

In total, 14 experts were interviewed. Descriptive data for the participants are presented in Table 1.

**TABLE 1:** Descriptive data for participants.

**Participant ID**	**Profession and qualifications**	**Place of work**	**Years of SUD expertise**	**Mental health expertise**	**Interventions used**	**VR familiarity**	**VRT familiarity**
1	Counselor (MBACP accredited)	Outpatient mental health treatment services (including dual diagnosis)	20	Yes, as a mental health Support Counselor	Counseling & Support, Psychosocial Intervention	No prior VR experience but aware of its workings	No experience with VRT delivery, but aware of its use in clinical practice
2	Psychotherapist, Counselor (MA, MBACP accredited)	Outpatient substance abuse treatment services & private practice (substance use disorder)	14	No	Medical detoxification, Cognitive Behavioral Therapy (CBT), Motivational Interviewing (MI), Psycho-dynamic approach	No prior VR experience and not aware of its workings	No experience with VRT delivery and not aware of its use in clinical practice
3	Clinical Psychologist	Outpatient and inpatient substance abuse treatment services & NHS services, & private practice (dual diagnosis)	19	Yes, as mental health, Think Family Practitioner	CBT, Think Family Approach (TFA), Eclectic Therapy Approach	No prior VR experience and not aware of its workings	No experience with VRT delivery and not aware of its use in clinical practice
4	Counselor (PGCert, MA, MBACP accredited)	Outpatient substance abuse treatment services & private practice (dual diagnosis)	11	Yes, as a mental health Therapist	Counseling, CBT, Mindfulness Based Intervention (MBI)	No prior VR experience and not aware of its workings	No experience with VRT delivery and not aware of its use in clinical practice
5	Clinical Psychologist	Outpatient substance abuse treatment services (dual diagnosis)	9	Yes, as a Clinical Psychologist	CBT	Prior VR experience in a non-clinical context	No experience with VRT delivery, but aware of its use in clinical practice
6	Counseling Psychologist (HCPC)	Private practice (dual diagnosis)	4	Yes, as a Psychologist in NHS & mental health treatment services	Person-Centered Therapy (PCT), CBT, Cognitive Analytic Therapy, Dialectical Behavior Therapy	No prior VR experience and not aware of its workings	No experience with VRT delivery and not aware of its use in clinical practice
7	Psychotherapist, Counselor (MSc, MBACP accredited)	Private practice	6	No	CBT, MI, PCT, Hypnotherapy	Prior VR experience in a non-clinical context	No experience with VRT delivery, but aware of its use in clinical practice
8	Psychotherapist (MA, PGDip)	Private practice (substance use disorder)	20	No	Internal Family Systems, Neuro-Linguistic Psychotherapy	No prior VR experience and not aware of its workings	No experience with VRT delivery and not aware of its use in clinical practice
9	Psychotherapist, Counselor (MBACP accredited, PGDip, MA)	Private practice (dual diagnosis)	4	Yes, as a Psychotherapist	Transactional Analysis	Prior VR experience in a non-clinical context	No experience with VRT delivery, but aware of its use in clinical practice
10	Specialist Senior NHS Nurse, Teaching Fellow	NHS services, (dual diagnosis)	30	Yes, as a Specialist Senior NHS Nurse and Teaching Fellow	CBT, MI, Cognitive Behavioral Family Therapy	Prior VR experience in a non-clinical context	No experience with VRT delivery and not aware of its use in clinical practice
11	University Professor	University (research into substance use and addictions	25	No	Opioid Substitution Therapy	No prior VR experience and not aware of its workings	No experience with VRT delivery and not aware of its use in clinical practice
12	Clinical Psychologist	Outpatient substance abuse treatment services (dual diagnosis)	26	Yes, as a Clinical Psychologist	MI, Behavioral, Contingency Management, Trauma	No prior VR experience but aware of its workings	No experience with VRT delivery, but aware of its use in clinical practice
13	Social work team leader (BA)	Outpatient substance abuse treatment services (substance use disorders)	5	No	TFA, Counseling, Medical intervention	Prior VR experience in a non-clinical context	No experience with VRT delivery, but aware of its use in clinical practice
14	Recovery support worker	Outpatient & inpatient substance abuse treatment services	4	Yes	12-Step, CBT, Contingency Management	No prior VR experience and not aware of its workings	No experience with VRT delivery and not aware of its use in clinical practice

### Themes

The identified themes mapped to: the safety of the exposure (1); the realism of the therapy (2); the insight VRT can offer into the condition and the therapeutic experience for the practitioners and the individuals in recovery (3). Each theme is divided into sub-themes which are associated with both positive and negative aspects of VRT and are presented in Table 2.

**TABLE 2:** Themes and sub-themes of the thematic analysis.

**Theme**	**Sub-theme**
Safety of VRT	Ecological Validity of VE Addictive Behavior Exploration Relapse Prevention Risk of relapse Risk of traumatizing Concern for early exposure to triggers Overconfidence risk Eligibility of individuals with comorbid mental health issues Mitigating measures
Realism	Avatar use Olfactory augmentation Personalization of cues Exposure mode Concern for engagement Concern for realistic representation
Additional insights for practitioners and individuals in recovery	Better understanding of individuals in recovery Monitoring of virtual behavior Adoption of a different viewpoint

The consensus view was that VRT would be suitable for recovery from any substance and for adults of any age. VRT was viewed as being particularly attractive to young people, due to their regular use of technology. Two participants, focusing on alcohol abuse, suggested that problematic drinkers or people whose abuse stems from psychological dependence could benefit more from VRT than people with severe alcohol dependence whose physical health would be the sole priority. All participants recommended the use of VRT as an assistive tool rather than a stand-alone intervention, as they believed it to be more useful for addressing symptoms than the underlying cause of the disorder. It was additionally underlined that to attend to the diverse needs of each individual, at each recovery stage, combined therapies would be required in any case.

#### Theme 1: Safety of VRT

This theme addresses the issue of safety in the use of VRT in SUD treatment. The ecological validity of the VE was believed to offer realistic and safe exposure to triggers, without the risk of immediate relapse. This was seen to be useful for individuals with SUDs to explore their addictive behavior and practice ways to prevent relapse. However, the risks of subsequent relapse, of traumatizing and of overconfidence about coping with triggers were raised. Early exposure to triggers and the suitability of VRT for people with comorbid mental health issues were also discussed. Mitigating measures were proposed to facilitate safety during VRT.

##### Ecological validity of the VE

The key element of VRT’s therapeutic value was collectively acknowledged to be the virtual representation of cues, rendering immediate relapse impossible and the exposure safe. “You’re allowing people to experience in a risk-free environment so there’s no immediate access to alcohol” (P1). Access to daily, triggering VEs was seen as valuable for therapy. “It will be a massive aid. Because it will be recreating a scenario that could be of an everyday, any day occurrence in their life. So we can see how they react to that, look at it differently” (P14). Simulating and tailoring situations that were hard to find in the real world, including embodying a specific individual via an avatar, were additionally thought by participants to offer a variety of triggering situations. “I think the good thing about VR also, apart from bringing it all together is, if they can manipulate aspects of it that might not be possible to manipulate. Having things that are really hard to happen in the real world” (P12).

##### Addictive behavior exploration

As VRT has the potential to provide safe exposure to diverse situations, participants believed that it could allow individuals to explore their triggers and consequent responses. “You can gain more information on what they are thinking. It will help them understand what their triggers might be” (P14). VRT could also be used to assess the individual’s openness about their substance abuse issue and facilitate a discussion about their triggers. One participant, referencing the Prochaska and DiClemente stages of change model (Prochaska et al., 1992), thought that it could assist prior to change (Pre-contemplation) or while considering change (Contemplation) by exploring and evaluating the individual’s addiction state. “I could see it being used with pre-contemplative people to explore the issues in their life that let them be pre-contemplative of change. I could see it being helpful when they’re planning for change” (P10).

##### Relapse prevention

Participants frequently mentioned the potential for VRT to help with relapse prevention. VRT was considered a safer alternative to *in vivo* ET, as it provided an environment in which people could learn to handle cravings, without exposing them to real life contact with the addictive substance. “Most people don’t ever learn that cravings don’t last forever. And learning that, in the virtual world would be really helpful to practice that *in vivo*, you know, this is ideal. But, the next thing they know, they’re drinking a pint of lager” (P10). The control VRT provided was also seen important by a number of participants for practicing coping skills. Firstly, individuals controlling their cravings virtually was thought to help them realize that they could perform this in reality, too, while experiencing in practice a positive feeling linked to coping. “It is about coping skills empowerment, recognizing, that they still have a choice…That they are powerful enough to refuse.” (P14). Secondly, individuals being able to control navigation and virtual interactions was viewed as positive for increasing their self-confidence while avoiding actual relapse: “People might gain in confidence and self-esteem about being, you know, in control of their lives. This might be one area that they have got control” (P10). VRT was additionally perceived by a few participants as beneficial for reintegration of people who have finished in-patient therapy and need to adjust to daily trigger exposure. “It can be quite a bubble when you’re in therapy, then they go back out into the real world almost, it’s quite a big jump. So it would be useful to have it as a tool” (P2). One participant recommended VRT as suitable for acting as a reality check for people who had been abstinent for years, since beliefs that they had been ‘cured’ and that they could therefore use again were common. Finally, using VRT as homework was suggested by a participant, as it could provide an extra layer of support and involvement. “This would be like a tool to assist a person, particularly in between therapy sessions” (P7).

##### Risk of relapse

Despite the relative safety of the VEs in terms of exposing individuals to triggers, participants felt strongly that VRT involved its own risks. Whilst there was no risk of relapsing during a VRT session, there may be a risk following a session, as they would have been exposed to immersive, tempting situations. “If I exposed somebody to an addiction experience, they’re regressing to a child ego state which is where their biggest vulnerabilities lie, but also the least reasonable decision making” (P9). However, participants acknowledged that for VRT to be therapeutically beneficial, it had to be sufficiently triggering. “What you want for the system to work, for me, has to be very arousing for them.” (P2). It was also acknowledged that the risk of relapse would remain regardless of mitigating measures, but that would be the case for any therapy. “We’re exposing them to potential triggers that they face in the real world, that’s kind of part of the process of therapy. I think it’s an acceptable risk” (P5). One participant suggested that if VRT were delivered in residential treatment, this risk would be reduced.

##### Risk of traumatizing

Most participants identified the risk of traumatizing an individual, by virtually exposing them to cues that they might not be ready or prepared to handle. “You’ve got the potential of traumatizing someone or re-traumatizing them, it’s almost a trauma exposure therapy” (P9). One participant noted that this would also be an issue for people with co-occurring mental health issues, as often an existing trauma would underpin their mental health distress and substance abuse. “Maybe post-traumatic stress disorder as both a cause of that mental health distress, but also a cause of their substance misuse, will be very, very common. Using virtual reality in an area before they were ready could be really detrimental” (P10).

##### Concern for early exposure to triggers

Participants were also concerned about whether people could cope with exposure to triggers before achieving abstinence or stability in use. “Certainly wouldn’t be doing it with someone who is dependent at drinking or drinking at increased levels of risk. I think it would be too difficult to manage earlier on” (P2). One participant reflected on whether exposing individuals to triggers at an early stage of recovery would be ethically problematic, but acknowledged that exposure would happen anyway in the everyday environment of individuals. “Whether you are doing this early on in the process raises up an ethical issue…I have to say that in their environments they are surrounded by those cues anyway” (P11). Readiness for VRT was considered by all participants to depend on their individual capacity. “I wouldn’t give it a time, I would give it in terms of the person’s progress to treatment and other resources available to them. They need to be ready to be built up” (P12).

##### Overconfidence risk

Some participants identified the risk of people feeling overconfident and falsely assuming that they were cured after managing the virtual, tempting situation. “It could give people a false sense of security. I’ve done my virtual reality thing. And I didn’t crack up. So now I can be on the real street corner” (P8). A number of participants also felt that individuals might overestimate their progress during therapy. “People have a tendency to think they’re doing better than they are.” (P8). For that reason, a few participants suggested that it would be useful for individuals to test their coping skills in real, correspondingly tempting situations after practicing them virtually, “They’ve still got to go in the real world and do it.” (P7).

##### Eligibility of individuals with comorbid mental health issues

Participants agreed that service users with mental health difficulties should not be excluded from VRT as they represent a major part of the SUD population in recovery. “…over 80 percent of everybody who attends for some kind of substance misuse treatment in this country has an identified mental health problem.” (P10). However, some expressed concerns about its safety for this population. They felt that for individuals with disorders connected to reality, disassociation and self-awareness, or individuals with hallucinations or psychosis, VRT could potentially be distressing or confusing. “If they are immersed in this exercise, they may not necessarily be aware of when that reality finishes and when it starts.” (P6). Another participant considered whether VRT implied that escaping reality is acceptable to people who try to mentally escape reality or avoid social interactions. “It may be that somebody came to this therapy and what they spend most of their time doing is escaping, then I put a headset on them do then I collude with their idea, that non-reality is more useful than reality?” (P9).

Participants thought that when individuals had anxiety or depression, their eligibility should be dependent on their capacity due to concerns that VRT might increase anxiety, if it were unfamiliar. “There is the possibility that this experience could heighten someone’s anxiety. There’s a sense of apprehension with that maybe. I think that’d be for person to person” (P9). The possibility of the VR headset distressing claustrophobic individuals was acknowledged by a few participants. “I think a claustrophobic person probably would not do well with that.” (P6). As such, a familiarization exposure to a neutral, daily scene was believed by all participants to promote a feeling of safety. “Obviously that’s useful for people who don’t know anything about virtual reality, or computers to feel safe that may help in some of those situations where we talk about anxiety… And it might be that people further up this scale may actually be able to have more access to that” (P9).

##### Mitigating measures

Other mitigation measures recommended by participants included proper assessment, preparation and a solid intervention plan. “If you used it too early or you didn’t assess properly you could actually send somebody right back, and there is not enough formulated approach” (P12). Reintegrating the individual after VRT emerged as the most important measure against relapse and as an ethical duty of practitioners. “What you don’t want them to do is actually they’ve watched this, then all of a sudden the cravings are through the roof, and they’re just gonna walk out of here and go straight to the pub. And I don’t think that’s ethical.” (P2). A trusting relationship between the individual in recovery and the practitioner was thought necessary for informed clinical decision-making. “I think when you’re using anything like this, it’s about knowing your client and your client knowing you. I can know when they’re becoming overly stimulated” (P2).

Finally, most participants emphasized that a skilled practitioner should guide the VRT sessions. A clinical psychologist or a Cognitive Behavioral Therapist were considered the most appropriate. For people with co-morbid mental health issues, practitioners who would normally deliver the daily treatments were seen as most appropriate, working in cooperation with the clinical supervisor and the medical professional attending the individual. “I’d talk about it with clinical supervision. And I’d probably run it by the person’s responsible medical, mental health professional” (P10). Participants therefore felt that with careful delivery, the risks accompanying VRT could be reduced. However, one participant compared the risk of VRT to that of PTSD treatment, noting that both would involve exposure to prior trauma that might worsen an individual’s state in the short term, but would prove therapeutic in the long term. “So, while someone might struggle with that and feel like they get a lot of cravings and thoughts about drinking, that’s an important and useful process for therapy. That’s what therapy is about.” (P5).

#### Theme 2: Realism

This theme addresses the level of realism involved in the use of VRT in SUD treatment. Elements that could influence the realism of the VEs were believed to be the use of avatars, the olfactory augmentation of the VEs, the personalization of cues and the exposure mode to the cues. Concerns were raised about the engagement of individuals with the exposure material and whether cues would be realistically represented.

##### Avatar use

Another aspect of VRT considered valuable by participants was the level of realism it could offer. Virtually exposing individuals to triggering situations was thought to be more realistic than imagining or discussing them. “Often in a residential treatment center, people will talk about certain situations, but it’s theoretical until you actually place them back in that situation, being on a street corner and knowing that the dealers will walk past. If we can virtually put them on that street corner, we can re-trigger and help them cope with what the physiology is like in those times” (P8). Including interactive avatars in the VEs was thought to have the potential to enhance realism. Participants believed that avatars could contribute to a more realistic version of role play. “So, what we would do is practice at role play about how you say no. And we would just kind of act out. VR could do that role. Probably, you know, more realistically” (P5). They thought that avatars simulating specific individuals who might apply social pressure in certain environments could contribute to people in recovery better handling cravings caused by these scenarios. This was seen by some participants as more appropriate for alcohol and nicotine abuse, as these are more socially acceptable than other substances and frequently linked to socializing and bonding. The ability to realistically practice the exact words required to refuse using was regarded valuable, especially in early abstinence. “In the early days, you kind of need to practice what you’re going to say to people. So, I think avatars would be really important in that, we don’t exist in isolation do we?” (P2).

Participants also suggested that interactive avatars could allow simulation of common, emotional scenarios to be used for craving management, increasing realism. Positive or negative emotional states were considered the real triggers for relapse and the actual cause of the addictive behavior, as substance abuse is often associated with the inability to regulate emotion. Simulating such scenarios was thought to help in that regard by evoking emotional, cognitive and behavioral reactivity while offering exposure to realistic triggering situations. “What we know about substance misuse is that most people use it to kind of manage emotions. And being exposed to an argument or an emotional trigger, would be also a part of relapse prevention. So it’s just making it more realistic” (P5). There were a range of interpersonal scenarios that were thought to be useful to simulate during VRT, including family settings and arguments or domestic abuse incidents. “Emotional states and particularly post-argument or post-conflict is a time where people begin to get real urges, to take away the feelings surrounding that conflict.” (P10). Participants believed that family-related scenarios could be used for family work and to educate people on the behavioral aspect, especially in group therapy. “For more in like group settings, you could use it to really like educate people and particularly more as a set for families” (P13).

##### Olfactory augmentation

It was thought that olfactory augmentation of the VEs with substance-related scents could further promote immersion and realism. Participants were largely positive that smell would assist in initiating craving in nicotine, alcohol or cannabis abuse. “The smell, that is what drives you mad. It makes the experience much more real” (P1). Some participants thought that olfactory augmentation and live props could overwhelm some individuals, associating immersion with stimulation and the likelihood of relapse. “It just depends on the individual. But, the scent might be a little bit too much. It’s making it very real then” (P13). It was recommended that each stimulus should be introduced progressively. “It is about pacing I think, isn’t it?” (P12). One participant mentioned that it would be useful to adjust the scent’s intensity. “It would be nice if you could play around with it, have some sort of levels” (P6). It was concluded by all participants that with consent of the individual in recovery, making the exposure as realistic as possible would be more beneficial. “With consent then anything that makes that experience more real the better” (P9).

##### Personalization of cues

Personalization of the cues was perceived as an additional means of ensuring realistic exposure. Participants explained that substance abuse habits would differ from individual to individual and personalizing the visual and audio cues would make the scenes more realistic and engaging. “Somebody dependant on smoking heroin, there is no point showing him syringes” (P11). Personalizing the avatars and their voices would also promote therapeutic involvement. “A woman might find another woman to keep asking her to drink more pressurizing” (P12). There was a view that it was important to avoid the avatar accidentally resembling a person who the individual in recovery may find traumatic, since substance abuse usually entails a prior traumatizing experience. “A male arriving in a pub. for a female is triggering in a whole different way that you didn’t envisage. That’s why I was talking about that control element of a VR experience” (P5).

##### Exposure mode

The ability to repeatedly expose individuals to identical VEs and scenarios was another feature discussed by participants. On the one hand, participants found the option to manipulate the scenario visited in a VE (in either random or controlled ways) appealing, as this would be more like real life. “I think that VR would be great, helpful for mopping up what was unexpected that’s where it has strengths, that it can be unexpected” (P12). It was suggested by some participants that changes could prevent desensitization to the scene. “I think minor changes would make it more realistic. I think that element of surprise in that first exposure. It hits that emotional level more.” (P2). This was thought by most participants to better prepare people for handling change, helping to ensure that their coping techniques would be transferable to other situations. “A lot of people who have addiction problems really struggle with change. So it’s almost learning to transfer the skills. So, when they go out into the real world and they hit that trigger, it’s all right, because I’ve practiced this and I’ve done it in 10 different scenarios, and it’s all been okay.” (P2).

Participants also thought that repeated exposure to the same scenario could be useful for building resilience. “I guess it depends on how well they performed on that scenario in previous attempts. you really need to get that response before moving on to a different scenario.” (P11). Some participants thought consistency of the exposure material was important to start with, so that individuals could track their progress. “Consistency would be better initially. So when you first looked at that you had this reaction but now you’re having this reaction.” (P8). Some participants mentioned that exposure to the same scenario would be helpful for training people to cope with triggers and achieve greater resilience as their reactions would depend on their corresponding emotional state. “I will do it again a few times. Because it depends on how the person is feeling.” (P14).

##### Concern for engagement

Despite the potential for realistic exposure to triggers, some participants were unsure whether genuine responses could be elicited. “If I found that it was very convincing in the sense that you could see that people were having genuine reactions, as opposed to playing with a toy then I can see it becoming very useful” (P8). They were concerned that recreating individuals, verbal interactions and tempting situations, like a friend overdosing, would be challenging. “It might be encountering a situation that brought you to use, being stressed, things which are quite difficult to mimic I would have thought within the virtual environment” (P11). A few participants doubted whether feelings could be induced artificially by fake cues, but also considered the artificial triggering of emotions unethical. “You can’t invoke those feelings artificially. They are happening in some part of the brain that is very fundamental and it is difficult to know how a fake cue can elicit real emotion. And it would be very unethical too.” (P11).

##### Concern for realistic representation

Another concern of a small number of participants was whether the software would be sophisticated enough to allow for realistic personalization of the VEs and avatars. “Because you won’t be able to completely personalize it, will you? To the extent that you can completely build your own, you know like in Minecraft?” (P5). One participant thought that personalized avatar appearance combined with drug abuse could result in confusion as to whether the individual was interacting with the real person or their avatar, and therefore suggested that only avatar behavior (and not appearance) should be personalized. “I think that because of how certain drugs can affect the mind, …if they were personalized to the characters’ [appearance] in real life, as opposed to adopting behaviors of characters in real life, then that person may cross over the boundary of what is reality and what is not.” (P1). The ethics of involving real people by virtually simulating them was registered as a concern by another participant. “To what extent are you including other human beings when they’re actually avatars?” (P8). Regardless of whether realistic personalization was achieved, it was thought by a few participants that virtual exposure to the addiction cues could still prove therapeutically valuable. “Just seeing that cigarette and just seeing that alcohol is a trigger. So, you’ve got part of the experience there, at least. And that’s still valuable” (P9).

#### Theme 3: Additional Insight for Practitioners and Individuals in Recovery

This theme addresses the additional insight that the use of VRT in SUD treatment can offer to both practitioners and individuals in recovery. Virtual exposure to triggers was believed to facilitate better understanding of individuals in recovery. Practitioners using a monitor to observe the virtual behavior of users was thought to increase further the understanding of individuals in recovery but was also linked to misconceptions about the individual’s responses and biased behavior. Adopting a different viewpoint via an avatar was perceived to be useful for individuals in recovery to gain insight into their addiction but the risks of relapse and disengagement were also associated with it.

##### Better understanding of individuals in recovery

Participants believed that VRT could offer insight into how individuals in recovery process triggering situations, either pre- or post- lapse, and that this could inform their subsequent intervention. “I f they relapse… you could kind of use it as a tool. Put this on [the VR headset]. Where did you go? Were any feelings that you are feeling now there last night? Let’s explore them. So, it could be a, you know, a really good tool…to get a bit more of a better understanding” (P3). Some participants additionally felt that it could be beneficial to deliver VRT within a group as their awareness about their substance abuse could be increased via sharing their experience. “I think discussing it as a group session would be even better. I think it would be really good that all ’d be kind of discussing how they feel about it, to be able to mirror it to the group” (P3). Olfactory augmentation of the VEs was similarly thought by participants to give greater insight into the root cause of the substance abuse issue. They stated that smell is one of the most powerful senses, able to trigger the surfacing and subsequent processing of memories of prior life events that led to the addiction initially. “Smell can really take you back it gets into where it needs to be. To the root of the problem quicker” (P2). Participants also appreciated the opportunity for interaction during the session, which might also support a better understanding. “And, so that’s kind of, live feedback, which is going to be useful for therapy” (P5).

##### Monitoring of virtual behavior

Using a monitor to observe the individuals was seen by most participants as an opportunity to improve understanding of the individual and their triggers. “We can look at what they are seeing, what they are reacting to and then we can gauge that and why they might have reacted to that and not to that” (P14). A monitor would also facilitate proper supervision, control over the exposure material and flow and help with clinical decision making. “I want to be in it as well, and just see what they see, so as to be sure when I’m talking to them about it or introducing it to them.” (P12). Participants suggested that it would increase practitioners’ interaction with the individuals, as they could comment on and discuss the individual’s virtual behavior. “You would be able to have a visual representation of it. And you can maybe ask additional questions based on what they’re seeing” (P10). Finally, a participant mentioned that it would demonstrate the practitioner’s involvement and attention to the therapeutic process, empowering the therapeutic alliance. “You’d want them to know that you were concerned and interested in what they were doing” (P8).

Although participants expressed a desire to observe the individuals’ actions, they were concerned that this might bias behavior, due to individuals being aware that they are observed by their therapist. “There’s something interesting about, does that person know, that you’re watching? Does that change how addiction works?” (P9). Lastly, some participants considered the possibility of inaccurate interpretation of the individual’s actions, as practitioners might focus on observing the monitor rather than other informative sources such as body language or verbal feedback from the individual. “I think I would just prefer to watch and actually get their feedback on what it is that they’re seeing, because we might think that’s what’s triggering them. But actually it’s the old bag in the side of the corner that reminds them of their Grandad” (P2).

##### Adoption of a different viewpoint

Adopting the viewpoint of another person had the potential to offer further insight to individuals in recovery, particularly for family work and psycho-education. Participants believed that by impersonating another member of their family through an avatar (change of perspective), which their practitioner or themselves would control, the individual may be able to perceive their view more clearly and have a direct experience of the impact of their substance abuse. “Understanding how all these perceive you when you’re drinking, if you can then look at how your child sees it. I think that’d be really powerful” (P2), “The reasoning of all that objective perspective, here’s what this person’s seen” (P7). Adopting the viewpoint of an avatar of the opposite sex was mentioned as having the potential to assist in addictive behavior exploration from a psychosocial approach. “A man, white English male, goes to the pub. his friends start drinking 20 pints. What happens if he goes in there as a woman? Does he have the same experience?” (P9).

Regardless of the insight it could offer, a few participants noted that the impact of adopting another viewpoint remains unexplored. “I don’t think there’s much precedent for knowing what. type of change that would create inside the person.” (P9). It was thought that experiencing their projected self virtually, being represented by a separate avatar, through the “eyes” of another person (via the currently adopted, avatar perspective) could increase self-shame, leading to cortisol production and, in turn, the urge to use. “You’re dad and you’ve been arguing with your wife because you’ve drunk too much and the son is upset. To put yourself in the son’s shoes. We might be setting this person up for shame” (P9). A participant also stated that the individual could disengage or terminate therapy due to being confronted, if this is not approached carefully. “So why would you have to do it really safely? You wouldn’t want to leave the person thinking, you know, now I’m being criticized. It might just cause them a reason for resistance and conflict with you” (P10).

## Discussion

Practitioners and researchers participating in this study had not previously delivered VRT as part of their SUD interventions. Nevertheless, some of their recommendations on the acceptability of VRT in SUD treatment align with conclusions drawn from trials of its use. For example, they felt that VRT should operate as an adjunct to other treatments; recent reviews of VRT SUD studies have similarly suggested that more controlled trials are needed to determine its efficacy as a stand-alone treatment (Trahan et al., 2019; Segawa et al., 2020). Additionally, most studies paired VRT with other interventions when evaluating it, achieving better results than studies which used it as a stand-alone intervention (Trahan et al., 2019; Segawa et al., 2020). VRT has also proved more effective when combined with CBT (Trahan et al., 2019; Segawa et al., 2020). Participants noted that the nature of VRT enables behavior training and that a CBT practitioner would be suitable for delivering it. Additionally, VRT delivered in a group was thought to potentially increase efficacy. This has been investigated in one study, which showed that group-based CET combined with VR in Alcohol Use Disorder (AUD) resulted in decreased craving (Ghiţă and Gutiérrez-Maldonado, 2018). Other literature also indicates that group treatments are slightly more effective than individual ones in maintaining abstinence (Lo Coco et al., 2019). Previous studies about technology-based treatments (web-based, online and artificial intelligence-based) further suggests that practitioners endorsed their use as an adjunct to substance abuse treatments, but not as stand-alone therapies (Quaglio et al., 2017).

Participants also drew on the effect that the presence of the practitioner would have on the delivery of VRT. On one hand, they suggested that being present during the VRT session and monitoring the individuals’ actions within the VEs could bias behavior. However, participants also mentioned this feedback would be important in informing their intervention and, in turn, in increasing the effectiveness of VRT. In addition to this, participants felt that being present during VRT could moderate the risks associated with the exposure. For instance, they believed that it would help them identify when the individuals would become overwhelmed and adjust the intensity of the VRT session, minimizing the risk of relapse. They also added that they could offer personalized debrief after the virtual exposure and address further the risk of subsequent relapse. Correspondingly, the importance of the practitioner’s presence has not yet been investigated in the context of Augmented Reality (AR)-based interventions for substance abuse treatments, but, like VRT, the clinical potential of flexible exposure to triggers are considerable (Vinci et al., 2020).

Previous experience of VR in any context did not seem to influence participants’ opinions about VRT in SUD treatment. Participants who had not tried VR before expressed the same concerns and identified the same risks and benefits of VRT as those with prior VR experience. However, participants who were familiar with VR recognized more technical challenges involved in VRT’s delivery. Although all participants suggested that personalization of the VEs would be necessary for a realistic and engaging outcome, those who had used VR previously acknowledged that building a VE from scratch would be challenging, requiring sophisticated software.

The combination of realistic and safe exposure to triggers offered by VRT was the reason that participants considered it as a good alternative to IE and CET for SUD treatment. The capability to simulate realistic tempting situations with multi-sensory feedback was believed to have the potential to enhance therapy when any type of cue interactivity was needed (e.g., with avatars or objects). Simultaneously, the fact that cues are virtual prevents actual exposure to the addictive substance and immediate relapse, ensuring safety in that regard. The option offered by VRT to control and personalize the cues also appealed to participants. Whether VRT can result in better therapeutic outcomes than IE or CET has not been examined yet within SUD studies (Martin et al., 2010; Hone-Blanchet et al., 2014; Amista, 2017; Bordnick and Washburn, 2019; Grochowska et al., 2019; Pericot-Valverde et al., 2019; Trahan et al., 2019; Wang et al., 2019; Worley, 2019; Kim and Kim, 2020; Segawa et al., 2020), but its realism, safety and the flexibility it offered in the presentation of the environmental content were thought to be positive for its potential in diverse recovery stages and contexts. However, there were also concerns that realism could also raise the risk of subsequent relapse by over-stimulating individuals. To avoid this, participants suggested that the individual capacity of each individual should be considered throughout. For this reason controls over the exposure material and flow (such as personalization or monitor use for observation) emerged as requirements, as well as benefits. Debrief after VRT was perceived as the most important safety measure and a formulated delivery the second most important one.

The use of avatars, controlled by either the practitioner or the individual in recovery, during VRT was perceived to be useful for a more insightful and realistic intervention. The ability to adopt a different viewpoint via an avatar (change of perspective) was considered powerful, but relapse or conflict with the practitioner was the risk if this was not managed carefully. Whilst the impact of changing perspective has not been explored for SUD, controlling a personalized avatar (via embodiment) in eating disorder VRT has been shown to have a positive effect on patients’ cognitive perception of their bodies (de Carvalho et al., 2017; Riva et al., 2019; Irvine et al., 2020; Kim and Kim, 2020). Moreover, simulating emotionally involving situations with avatars as triggers and adopting a different avatar viewpoint, virtually, was seen by participants as beneficial for family or relationship-oriented work, as well as individual therapy. A study exploiting the use of a drug user’s avatar viewpoint in a virtual reality setting showcased that increased empathy was felt by participants in this condition, compared to the desktop setting, suggesting the therapeutic potential of adopting an avatar’s viewpoint for eliminating stigma and facilitating empathy (Christofi et al., 2020). However, a study of VRT for treating AUD found that observing a general argument scene to invoke lower craving than situations involving social pressure (Hone-Blanchet et al., 2014). This might mean that for emotional scenes to be triggering a level of personalization is required, as stated by participants in this study.

Participants believed that the target population most likely to benefit from VRT would be young people due to their frequent use of technology. This has not been examined in previous SUD studies as participants were all middle-aged (Trahan et al., 2019). A study of the acceptability of VR headsets in older adults found there were positive attitudes, especially after the first use (Huygelier et al., 2019), suggesting that age should be not considered a barrier. Similarly, studies about wearable and wireless mobile Health technologies reported that individuals were open to wearing and using such devices to monitor drug and alcohol use and related parameters for relapse prevention purposes (Goldfine et al., 2020). Moreover, a few participants were concerned that people with mental health issues relating to self-awareness might be confused about which reality they were in, or that people with severe mental health issues might become distressed. However, meta-analysis and reviews of VR studies with people with depression and schizophrenia showed positive results in reducing anxiety and depression symptoms and assisting in cognitive re-adjustment (Macedo et al., 2015; Fodor et al., 2018; Rus-Calafell et al., 2018; Zeng et al., 2018; Grochowska et al., 2019).

Participants further suggested that individuals should be delivered VRT when they feel prepared to handle exposure to triggers, after any medical issues caused by SUDs have been attended to and in co-operation with any medical professionals involved in their care, particularly if there is a comorbid mental health difficulty. Participants did not identify cybersickness – symptoms such as nausea, eye tiredness and, in rare cases, vomiting – as a particular problem for individuals with SUD despite the existence of linked conditions such as Mallory-Weiss syndrome which can lead to bleeding in the gastroesophageal junction after vomiting (Haber and Kortt, 2020). Other populations that might be impacted by the use of VR include individuals with epilepsy, especially if cybersickness is experienced. So far, studies about the use of VR for cognitive assessment and learning of epileptic patients have appeared promising, with no serious adverse effects reported (Cánovas et al., 2011; Rosas et al., 2013; Grewe et al., 2014; Maidenbaum et al., 2019). VR has been known to cause cybersickness on occasions when the frame rate is low and there is display latency, when the field of view is wider than 100 degrees and when the user moves quickly within the VE (Kim et al., 2020; Stanney et al., 2020). Other causes of cybersickness may relate to the postural stability, the eye movement and certain neural responses of an individual to the VEs (Kim et al., 2020; Stanney et al., 2020). Ultimately, most causes of cybersickness, such as the display rate, can be addressed by the design of the VR applications and by the use of modern VR headsets (Kim et al., 2020; Stanney et al., 2020).

### Limitations and Strengths

The participants were aware that they were being interviewed by a researcher focusing on the development of a VR application to be used in SUD treatment, which may have biased their responses. Moreover, the views of participants about the use of VRT in SUD treatment weren’t based on their clinical experience of VRT and, thus, these views might have differed if participants have delivered VRT as part of their SUD interventions. Furthermore, participants did not consider the impact that cybersickness may have on the delivery and efficacy of VRT.

The sample consisted of practitioners from private practices, treatment services including rehabilitation and integration services, charities, the NHS, who delivered SUD treatments, and academics who were involved in the design of SUD treatments, allowing different perspectives to emerge. Some participants specialized both in substance abuse and mental health treatment, offering an insight into the eligibility of dual-diagnosed individuals. Participants employed a wide range of interventions, including CBT, psychodynamic therapy, and systemic therapy. To date, this study is the only qualitative study examining experts’ opinions on the acceptability and potential of VRT for use in therapeutic settings.

Future research should involve clinical trials to explore aspects that might affect the efficacy of VRT, such as avatar use, olfactory augmentation, personalization of cues and the therapeutic context to be integrated. In these trials, recruitment of participants should be informed, except by the input of practitioners with an expertise in SUD treatment, by the input also of medical doctors and practitioners with an expertise in mental health treatment, so as to examine the suitability of VRT for different populations, forming safe delivery protocols. Cybersickness checks and any causes of cybersickness should also be part of future clinical trials. Future studies can further explore the acceptability of online VRT, with the individuals using either a standard VR headset or their smartphone attached to a VR headset case to run the VR application, and the practitioners to deliver the VRT session via a video call. The potential of AR to assist individuals’ recovery at their own homes, either as a self-help or during an online session with a practitioner, can also be explored. Monitoring of psychophysiological and behavioral responses through wearable technologies can additionally be investigated for complementing recovery and assessment of patients. Studies about the treatment preferences of individuals with different levels of substance abuse can also explore the acceptability of VRT as a potential treatment option, compared to the SUD treatments available. Finally, this and similar qualitative work about VRT’s delivery should be updated as close co-operation between researchers and practitioners could lead to meaningful clinical trials and precipitate the integration of VRT into SUD recovery.

## Conclusion

Practitioners and researchers recommended VRT’s use in SUD treatment as an assistive tool throughout recovery. The insight that VRT could offer during SUD treatment both to individuals in recovery and practitioners was acknowledged. VRT was thought suitable for any adult, including people with a mental health condition or trauma, providing that preparation, familiarization via exposure to neutral VEs and aftercare are performed. Realism, personalization, avatar use and the feature of changing the avatar viewpoint (allowing when relevant the individual to see themselves as a separate person), olfactory augmentation and an observation monitor for the practitioners were perceived as important factors in VRT’s efficacy and the therapeutic contexts it could serve. The individual in recovery feeling overconfident about coping and subsequently relapsing or being traumatized were identified as risks. The reported practitioners’ and researchers’ views are useful for informing future VRT applications in SUD treatment.

## Data Availability Statement

The original contributions presented in the study are included in the article/supplementary material, further inquiries can be directed to the corresponding author/s.

## Ethics Statement

The studies involving human participants were reviewed and approved by Ethical approval for the study was granted by the University of Manchester Research Ethics Committee (Ref.: 2019-6983-10288). The patients/participants provided their written informed consent to participate in this study.

## Author Contributions

RS designed the topic guide, performed the interviews, the transcriptions and the thematic analysis, and wrote the manuscript. Guidance on the topic guide design, transcription and thematic analysis were given by LG. Further feedback and validation on the topic guide were also provided by CJ and SP. Finally, feedback, validation and proofreading were provided by LG, CJ, and SP during the thematic analysis and the writing of the manuscript. All authors contributed to the article and approved the submitted version.

## Conflict of Interest

The authors declare that the research was conducted in the absence of any commercial or financial relationships that could be construed as a potential conflict of interest.

## References

[B1] Amista F. N. (2017). Trends and future of virtual reality for addiction treatment of substance use disorders a review of systematic literature. *J. Digital Contents Soc.* 18 1551–1560.

[B2] Barber S. Harker R. Pratt A. (2017). *Human and Financial Costs of Drug Addiction. House of Commons Library.* Debate Pack: Number CDP-0230. Available online at: https://researchbriefings.files.parliament.uk/documents/CDP-2017-0230/CDP-2017-0230.pdf (accessed April 27, 2020).

[B3] Bordnick P. S. Washburn M. (2019). “Virtual environments for substance abuse assessment and treatment,” in *Virtual Reality for Psychological and Neurocognitive Interventions. Virtual Reality Technologies for Health and Clinical Applications*, eds Rizzo A. Bouchard S. (New York, NY: Springer).

[B4] Braun V. Clarke V. (2006). Using thematic analysis in psychology. *Qual. Res. Psychol.* 3 77–101. 10.1191/1478088706qp063oa 32100154

[B5] Cánovas R. León I. Serrano P. Dolores Roldán M. Cimadevilla J. M. (2011). Spatial navigation impairment in patients with refractory temporal lobe epilepsy: evidence from a new virtual reality-based task. *Epilepsy Behav.* 22 364–369. 10.1016/j.yebeh.2011.07.021 21873120

[B6] Christofi M. Michael-Grigoriou D. Kyrlitsias C. (2020). A virtual reality simulation of drug users’ everyday life: the effect of supported sensorimotor contingencies on empathy. *Front. Psychol.* 11:1242. 10.3389/fpsyg.2020.01242 32581979 PMC7289998

[B7] de Carvalho M. R. de Santana Dias T. R. Duchesne M. Nardi A. E. Appolinario J. C. (2017). Virtual reality as a promising strategy in the assessment and treatment of bulimia nervosa and binge eating disorder: a systematic review. *Behav. Sci.* 7:43. 10.3390/bs7030043 28698483 PMC5618051

[B8] DeJonckheere M. Vaughn L. M. (2019). Semistructured interviewing in primary care research: a balance of relationship and rigour. *Fam. Med. Commun. Health* 7:e000057. 10.1136/fmch-2018-000057 32148704 PMC6910737

[B9] Deng W. Hu D. Xu S. Liu X. Zhao J. Chen Q. (2019). The efficacy of virtual reality exposure therapy for PTSD symptoms: a systematic review and meta-analysis. *J. Affect. Disord.* 257 698–709. 10.1016/j.jad.2019.07.086 31382122

[B10] Fodor L. A. Coteţ C. D. Cuijpers P. Szamoskozi Ş David D. Cristea I. A. (2018). The effectiveness of virtual reality based interventions for symptoms of anxiety and depression: a meta-analysis. *Sci. Rep.* 8:10323. 10.1038/s41598-018-28113-6 29985400 PMC6037699

[B11] Ghiţă A. Gutiérrez-Maldonado J. (2018). Applications of virtual reality in individuals with alcohol misuse: a systematic review. *Addict. Behav.* 81 1–11. 10.1016/j.addbeh.2018.01.036 29421343

[B12] Ghiţă A. Hernandez-Serrano O. Ruiz J. Monras M. Ortega L. Mondon S. (2019). Craving and anxiety responses as indicators of the efficacy of virtual reality-cue exposure therapy in patients diagnosed with alcohol use disorder. *Annu. Rev. Cyberther. Telemed.* 17 77–82.

[B13] Goldfine C. Lai J. T. Lucey E. Newcomb M. Carreiro S. (2020). Wearable and wireless mHealth technologies for substance use disorder. *Curr. Addict. Rep.* 7 291–300. 10.1007/s40429-020-00318-8 33738178 PMC7963000

[B14] Gregg L. Tarrier N. (2007). Virtual reality in mental health: a review of the literature. *Soc. Psychiatry Psychiatr. Epidemiol.* 42:343. 10.1007/s00127-007-0173-4 17431528

[B15] Grewe P. Lahr D. Kohsik A. Dyck E. Markowitsch H. J. Bien C. G. (2014). Real-life memory and spatial navigation in patients with focal epilepsy: ecological validity of a virtual reality supermarket task. *Epilepsy Behav.* 31 57–66. 10.1016/j.yebeh.2013.11.014 24361763

[B16] Grochowska A. Jarema M. Wichniak A. (2019). Virtual reality – a valuable tool to advance treatment of mental disorders. *Arch. Psychiatry Psychother.* 1 65–73. 10.12740/APP/101654

[B17] Haber P. S. Kortt N. C. (2020). Alcohol use disorder and the gut. *Addiction* 116 658–667. 10.1111/add.15147 32511812

[B18] Hone-Blanchet A. Wensing T. Fecteau S. (2014). The use of virtual reality in craving assessment and cue-exposure therapy in substance use disorders. *Front. Hum. Neurosci.* 8:844. 10.3389/fnhum.2014.00844 25368571 PMC4201090

[B19] Huygelier H. Schraepen B. van Ee R. Abeele V. V. Gillebert C. R. (2019). Acceptance of immersive head-mounted virtual reality in older adults. *Sci. Rep.* 9:4519. 10.1038/s41598-019-41200-6 30872760 PMC6418153

[B20] Irvine K. R. Irvine A. R. Maalin N. McCarty K. Cornelissen K. K. Tovée M. J. (2020). Using immersive virtual reality to modify body image. *Body Image* 33 232–243. 10.1016/j.bodyim.2020.03.007 32408166

[B21] Kim J. Luu W. Palmisano S. (2020). Multisensory integration and the experience of scene instability, presence and cybersickness in virtual environments. *Comput. Hum. Behav.* 112: 106484. 10.1016/j.chb.2020.106484

[B22] Kim S. Kim E. (2020). The use of virtual reality in psychiatry: a review. *J. Korean Acad. Child Adolesc. Psychiatry* 31 26–32. 10.5765/jkacap.190037 32612410 PMC7324842

[B23] Lindner P. Miloff A. Zetterlund E. Reuterskiöld L. Andersson G. Carlbring P. (2019). Attitudes toward and familiarity with virtual reality therapy among practicing cognitive behavior therapists: a cross-sectional survey study in the era of consumer VR platforms. *Front. Psychol.* 10:176. 10.3389/fpsyg.2019.00176 30800086 PMC6376952

[B24] Lo Coco G. Melchiorib F. Oienid V. Infurnab M. R. Straussc B. Schwartzec D. (2019). Group treatment for substance use disorder in adults: a systematic review and meta-analysis of randomized-controlled trials. *J. Substance Abuse Treat.* 99 104–116. 10.1016/j.jsat.2019.01.016 30797382

[B25] Macedo M. Marques A. Queirós C. (2015). Virtual reality in assessment and treatment of schizophrenia: a systematic review. *J. Brasil. Psiquiatr.* 64 70–81. 10.1590/0047-2085000000059

[B26] Maidenbaum S. Patel A. Stein E. Jacobs J. (2019). “Spatial memory rehabilitation in virtual reality – extending findings from epilepsy patients to the general population,” in *Proceedings of the 2019 International Conference on Virtual Rehabilitation (ICVR)*, Tel Aviv, 1–7. 10.1109/ICVR46560.2019.8994573

[B27] Maples-Keller J. L. Yasinski C. Manjin N. Rothbaum B. O. (2017). Virtual reality-enhanced extinction of phobias and post-traumatic stress. *Neurotherapeutics* 14 554–563. 10.1007/s13311-017-0534-y 28512692 PMC5509629

[B28] Martin T. LaRowe D. S. Malcolm R. (2010). Progress in cue exposure therapy for the treatment of addictive disorders a: review update. *Open Addict. J.* 3 92–101. 10.2174/1874941001003010092

[B29] McIntosh M. J. Morse J. M. (2015). Situating and constructing diversity in semi-structured interviews. *Glob. Qual. Nurs. Res.* 2:2333393615597674. 10.1177/2333393615597674 28462313 PMC5342650

[B30] Mellentin A. I. Skøt L. Nielsen B. Schippers G. M. Nielsen A. S. Stenager E. (2017). Cue exposure therapy for the treatment of alcohol use disorders: a meta-analytic review. *Clin. Psychol. Rev.* 57 195–207. 10.1016/j.cpr.2017.07.006 28781153

[B31] National Institute for Health and Care Excellence (2007a). *Drug Misuse in Over 16s: Opioid Detoxification. Clinical Guidance CG51.* Available online at: https://www.nice.org.uk/guidance/cg51/chapter/1-Guidance#formal-psychosocial-interventions (accessed April 27, 2020). 31825576

[B32] National Institute for Health and Care Excellence (2007b). *Drug Misuse in Over 16s: Opioid Detoxification. Clinical Guidance CG52.* Available online at: https://www.nice.org.uk/guidance/cg52/chapter/1-Guidance#specific-psychosocial-interventions (accessed April 27, 2020). 31825576

[B33] National Institute for Health and Care Excellence (2011). *Alcohol-use Disorders: Diagnosis, Assessment and Management of Harmful Drinking (high-risk drinking) and Alcohol Dependence. Clinical Guideline CG115.* Available online at: https://www.nice.org.uk/guidance/cg115 (accessed April 27, 2020). 39808037

[B34] National Institute on Drug Abuse (2018). *Principles of Drug Addiction Treatment: A Research-Based Guide (Third Edition).* North Bethesda, MD: National Institute on Drug Abuse.

[B35] Pericot-Valverde I. Secades-Villa R. Gutiérrez-Maldonado J. (2019). A randomized clinical trial of cue exposure treatment through virtual reality for smoking cessation. *J. Substance Abuse Treat.* 96 26–32. 10.1016/j.jsat.2018.10.003 30466545

[B36] Prochaska J. O. DiClemente C. C. Norcross J. C. (1992). In search of how people change. Applications to addictive behaviours. *Am. Psychol.* 47:1102. 10.1037/0003-066x.47.9.1102 1329589

[B37] Quaglio G. Schellekens A. Blankers M. Hoch E. Karapiperis T. Esposito G. (2017). A brief outline of the use of new technologies for treating substance use disorders in the European Union. *Eur. Addict. Res.* 23 177–181. 10.1159/000478904 28803249

[B38] Riva G. Gutiérrez-Maldonado J. Dakanalis A. Ferrer-García M. (2019). “Virtual reality in the assessment and treatment of weight-related disorders,” in *Virtual Reality for Psychological and Neurocognitive Interventions. Virtual Reality Technologies for Health and Clinical Applications*, eds Rizzo A. Bouchard S. (New York, NY: Springer).

[B39] Rosas K. Parrón I. Serrano P. Cimadevilla J. M. (2013). Spatial recognition memory in a virtual reality task is altered in refractory temporal lobe epilepsy. *Epilepsy Behav.* 28 227–231. 10.1016/j.yebeh.2013.05.010 23773979

[B40] Rus-Calafell M. Garety P. Sason E. Craig T. J. K. Valmaggia L. R. (2018). Virtual reality in the assessment and treatment of psychosis: a systematic review of its utility, acceptability and effectiveness. *Psychol. Med.* 48 362–391. 10.1017/S0033291717001945 28735593

[B41] Schwartzman D. Segal R. Drapeau M. (2012). Perceptions of virtual reality among therapists who do not apply this technology in clinical practice. *Psychol. Serv.* 9 310–315. 10.1037/a0026801 22867123

[B42] Segal R. Bhatia M. Drapeau M. (2011). Therapists’ perception of benefits and costs of using virtual reality treatments. *Cyberpsychol. Behav. Soc. Netw.* 14 29–34. 10.1089/cyber.2009.0398 21329440

[B43] Segawa T. Baudry T. Bourla A. Blanc J. V. Peretti C. S. Mouchabac S. (2020). Virtual Reality (VR) in assessment and treatment of addictive disorders: a systematic review. *Front. Neurosci.* 13:1409. 10.3389/fnins.2019.01409 31998066 PMC6965009

[B44] Simon J. Etienne A. M. Bouchard S. Quertemont E. (2020). Alcohol craving in heavy and occasional alcohol drinkers after cue exposure in a virtual environment: the role of the sense of presence. *Front. Hum. Neurosci.* 14:124. 32296322 10.3389/fnhum.2020.00124 PMC7136534

[B45] Singh S. Nathan-Roberts D. (2019). Virtual reality exposure therapy and military personnel with post-traumatic stress disorder: a systematic review. *Proc. Hum. Factors Ergon. Soc. Annu. Meet.* 63 1378–1383. 10.1177/1071181319631178

[B46] Stanney K. Lawson B. D. Rokers B. Dennison M. Fidopiastis C. Stoffregen T. (2020). Identifying causes of and solutions for cybersickness in immersive technology: reformulation of a research and development agenda. *Int. J. Hum. Comput. Interact.* 36 1783–1803. 10.1080/10447318.2020.1828535

[B47] Trahan M. H. Maynard B. R. Smith K. S. Farina A. S. J. Khoo Y. M. (2019). Virtual reality exposure therapy on alcohol and nicotine: a systematic review. *Res. Soc. Work Pract.* 29 876–891. 10.1177/1049731518823073

[B48] Vinci C. Brandon K. O. Kleinjan M. Brandon T. H. (2020). The clinical potential of augmented reality. *Clin. Psychol. Sci. Pract.* 27:e12357. 10.1111/cpsp.12357 33223628 PMC7673236

[B49] Wang Y. Liu M. Shen Z. (2019). A virtual reality counterconditioning procedure to reduce methamphetamine cue-induced craving. *J. Psychiatric Res.* 116 88–94. 10.1016/j.jpsychires.2019.06.007 31226580

[B50] World Health Organisation (2018). *World Drug Report 2018, United Nations Publication*, Sales No. E.18.XI.9. Geneva: World Health Organization.

[B51] Worley J. (2019). Virtual reality for individuals with substance use disorders. *J. Psychosoc. Nurs. Ment. Health Serv.* 57 15–19. 10.3928/02793695-20190430-01 31162622

[B52] Zeng N. Pope Z. Lee J. E. Gao Z. (2018). Virtual reality exercise for anxiety and depression: a preliminary review of current research in an emerging field. *J. Clin. Med.* 7:42. 10.3390/jcm7030042 29510528 PMC5867568

